# Predictors of climate change literacy in the era of global boiling: a cross-sectional survey of Egyptian nursing students

**DOI:** 10.1186/s12912-024-02315-y

**Published:** 2024-09-26

**Authors:** Mohamed Hussein Ramadan Atta, Mohamed A. Zoromba, Maha Gamal Ramadan Asal, Eman Sameh AbdELhay, Abdelaziz Hendy, Mervat Amin Sayed, Huwida Hamdy Abd Elmonem, Omnya Sobhy Mohamad El-ayari, Ibrahim Sehsah, Islam Sameh AbdELhay, Alzahraa Abdel Aziz Omar Abdel Rahman, Selwan Mahmoud Ibrahim Balha, Heba Mostafa Ali Taha, Hanady. Sh. Shehata, Ahmed Abdellah Othman, Ahmed Zaher Mohamed, Mahitab Mohamed Abdelrahman, Noha Mohammed Ibrahim Ibrahim, Eman Hassan Mahmoud Hassan, Hend Ali Mohamed Abd El-fatah, Amal AbdElaal Mohamed Ali, Mohamed Farag Awad Elsmalosy, Eslam Reda Machaly, Mohamed Adel Ghoneam, Amal Fawzy Zaki Ali, Mira Naguib Abdelrazek Elfar, Ahmed Abdelwahab Ibrahim El-Sayed, Marwa Fouad Hanafy Mahmoud, Eman Arafa Hassan

**Affiliations:** 1 Psychiatric and Mental Health Nursing Department, Faculty of Nursing, Faculty of Nursing, Psychiatric Nursing Department, Alexandria University, Admeon Freemon St, Semoha, Alexandria City, Egypt; 2 Psychiatric and Mental Health Nursing, Faculty of Nursing, Mansoura University, Mansoura City, Egypt; 3 Medical Surgical Nursing Department, Faculty of Nursing, Alexandria University, Alexandria, Egypt; 4 Pediatric Nursing, Faculty of Nursing, Ain Shams University, Cairo City, Egypt; 5 Community Health Nursing Faculty of Nursing at Fayoum University, Fayoum City, Egypt; 6 Pediatric Nursing, Fayoum City, Egypt; 7 Faculty of Nursing, Psychiatric Nursing and Mental Health, Kafr ElSheikh University, Kafr Elsheikh City, Egypt; 8 Faculty of Nursing, MTI University, Cairo City, Egypt; 9 Nursing Administration, Faculty of Nursing, Mansoura University, Mansoura City, Egypt; 10 Faculty of Nursing, Psychiatric Mental Health Nursing, Minya University, Minya City, Egypt; 11 Psychiatric and Mental Health Nursing, Faculty of Nursing, Tanta University University, Tanta City, Egypt; 12 Nursing Administration Department University, Assiut City, Egypt; 13 Family and Community Health Nursing, Faculty of Nursing, Menofia University, Menofia City, Egypt; 14 Nursing Administration, Sohag University, Sohag City, Egypt; 15 Faculty of Nursing, Psychiatric Mental Health Nursing, Ain Shams University, Cairo City, Egypt; 16 Faculty of Nursing, Suez Canal University, Ismailia city, Egypt; 17 Medical-Surgical Nursing, Faculty of Nursing, Port Said University, Port- Said City, Egypt; 18 Pediatric Nursing Helwan University, Helwan City, Egypt; 19 Maternity, Obstetrics and Gynecology, Faculty of Nursing, Suez Canal University, Ismailia city, Egypt; 20 South Valley University, Qena City, Egypt; 21 Psychiatric Mental Health Nursing, Matrouh University, Marsa Matroh City, Egypt; 22 Pediatric Nursing, Suez Canal University, Suez City, Egypt; 23 Critical Care and Emergency Nursing, Beni-Suef University, Beni-Suef City, Egypt; 24 Technical Institute of Nursing Zagazig University, Zagazig City, Egypt; 25 Psychiatric and Mental Health Nursing Department, Faculty of Nursing, Alexandria University, Alexandria City, Egypt; 26 Nursing Administration Department, Faculty of Nursing, Alexandria University, Alexandria City, Egypt; 27 Nursing Education Department, Faculty of Nursing, Damanhur University, Damanhour City, Egypt; 28 Critical Care and Emergency Nursing, Alexandria University, Alexandria City, Egypt

**Keywords:** Climate change, Egyptian, Global boiling, Literacy, Multi-site survey, Nursing students, Predictors

## Abstract

**Background:**

Climate changes have led to health and environmental risks, so it has become essential to measure climate change literacy among the entire population, especially nursing students. The significant role of nursing students in raising public awareness and future healthcare roles emphasizes assessing the predictors of climate change literacy among nursing students.

**Aims:**

This study seeks to identify the predictors of climate change literacy among nursing students in A Multi-Site Survey.

**Design:**

A multi-site descriptive cross-sectional study adheres to the guidelines outlined in A Consensus-Based Checklist for Reporting Survey Studies collected for five months, from the 1st of July 2023 to November 2023. The study participants comprise 10,084 nursing students from all 27 governments in Egypt. The researcher used the Predictors of Nursing Students’ Climate Change Literacy scale in this study. Data was collected, with 25 min average time to complete. Backward multiple linear regression was used to identify these predictors.

**Results:**

In the current study, nursing students demonstrated a moderate understanding of climate science (mean score 14.38), communication and advocacy skills (mean score 14.41), and knowledge of adaptation and mitigation strategies (mean score 13.33). Climate health impacts (mean score 17.72) emerged as the domain with the highest level of knowledge. No significant differences in climate literacy were observed across diverse student backgrounds (all p-values were > 0.05). Perceived faculty knowledge of climate change positively correlated with all four domains of climate literacy and emerged as a significant predictor in multiple linear regression analyses (all *p*-values were < 0.001).

Implication.

While our findings highlight significant predictors of climate literacy, it is essential to recognize that these results identify associations rather than causal relationships. Based on these associations, it is recommended that nursing professionals be equipped with comprehensive knowledge of climate adaptation strategies to better advocate for and implement effective public health measures.

**Supplementary Information:**

The online version contains supplementary material available at 10.1186/s12912-024-02315-y.

## Introduction

The escalating threat of climate change has emerged as one of humanity’s most pressing challenges in the twenty-first century. Its effects go beyond the environment, creating significant health problems worldwide and affecting many aspects of human life [1]. The World Health Organization (WHO) has highlighted the profound effects of climate change on health, including the increased prevalence of vector-borne diseases such as malaria and dengue fever, heat-related illnesses, respiratory problems due to air pollution, and food insecurity [2].

Climate change endangers ecosystems and directly impacts human health through various pathways [3, 4]. According to the Carnegie Endowment report about Egypt, characterized by its hot, dry climate and predominantly desert landscape, confronts escalating environmental challenges exacerbated by climate change [5]. These include extreme temperatures, erratic precipitation patterns, rising sea levels, land subsidence, coastal flooding, shoreline erosion, soil salinity, and persistent droughts. These interconnected impacts intensify water scarcity, threaten food security, displace vulnerable populations, and destabilize the economy. Given Egypt’s heavy reliance on the Nile for freshwater and its lifeline amid minimal annual precipitation, nearly all Egyptians reside in the narrow Nile Valley and Delta regions. This demographic concentration underscores the critical need for comprehensive research to mitigate these risks, address governance shortcomings, and bolster resilience in mounting climate pressures [1, 5, 6].

Nurses, the backbone of the healthcare system and frontline caregivers, play a vital role in mitigating these health impacts and promoting community resilience [7]. Their ability to educate patients, advocate for preventive measures, and manage climate-related illnesses is essential for safeguarding public health in a changing climate [8]. Therefore, enhancing climate change literacy among nursing students is paramount to building a more resilient healthcare system prepared to confront the complexities of a changing climate by equipping future healthcare professionals with the knowledge and skills necessary to address emerging health challenges [9, 10].

## Background

Climate change, defined by the United Nations Framework Convention on Climate Change (UNFCCC) as human-induced alterations to the Earth’s atmosphere, is a primary global environmental concern [1]. It has significant health implications, causing lasting changes in weather patterns worldwide [1, 10]. Although “global boiling” emphasizes extreme temperature rises, climate change is a more comprehensive phenomenon that involves various interconnected factors, such as changes in solar radiation, deforestation, and heightened greenhouse gas emissions. Together, these factors lead to altered weather patterns, rising sea levels, loss of biodiversity, and more frequent and severe climate-related events, impacting ecosystems and human societies worldwide [11, 12]. Its effects extend beyond individual health, threatening clean air, water, food, and shelter, leading to various health problems [11, 13]. Climate change is a complex issue impacting social, economic, political, geographical, ecological, and psychological aspects [10, 13, 14]. Its growing impact on human health and healthcare systems is a concern [15]. Egypt is especially vulnerable with its strained land and water resources [16, 17].

The intricate link between environmental factors and human health underscores the growing importance of climate change for nurses [18, 19]. Nurses can play a vital role by promoting environmentally sustainable and climate-resilient healthcare systems [20]. To do this effectively, they must understand how climate change alters health determinants and implement appropriate adaptation and mitigation strategies [17, 21]

The International Council of Nurses (ICN) emphasizes integrating sustainability and climate change education into nursing curricula [19, 22]. This will empower nurses to become leaders in building climate-resilient health systems [22, 23]. Additionally, advanced education is needed globally to equip future nurses with the skills to address climate change’s social, economic, and environmental consequences [24].

## Theoretical framework: the ecological systems theory

Urie Bronfenbrenner’s ecological systems theory offers a compelling lens through which nurses can address climate change. This theory asserts that individuals operate within interconnected systems, ranging from immediate environments (microsystems) to larger societal contexts (macrosystems) [25]. With its far-reaching implications, climate change underscores the interconnectedness of ecological and social systems characterized by complex dynamics [22]. The Intergovernmental Panel on Climate Change (IPCC) echoes this sentiment, advocating for a transformative approach to managing social-ecological systems (SES) [23]. Eriksen et al. (2021) and Yang et al. (2023) support this perspective, emphasizing the imperative to rethink strategies for addressing climate change and its impacts on SES [19] [21].

In nursing education, this framework highlights the interplay between personal attributes, educational institutions, and broader social influences that shape nursing students’ understanding of climate issues [26] [27–29]. This connection is pivotal in exploring predictors of climate change literacy among nursing students, as it underscores how educational environments and societal factors contribute to their comprehension of environmental challenges.

## Knowledge gaps and significance of the study

University education plays a crucial role in equipping future healthcare professionals. Understanding nursing students’ climate change awareness is essential for developing adaptation strategies and disaster risk reduction plans [30]. Existing research shows varying levels of awareness among nursing students. While some studies show positive attitudes toward climate change [31], others highlight inadequate knowledge about its health impacts and the need for more training [32, 33] In addition, Ofori et al., 2023 study about climate change knowledge, perception, and attitude found that students’ overall knowledge score on climate change was moderate and emphasized the importance of incorporating climate change science into the curricula at all levels of pre-tertiary and university education, for both science and non-science programs [34].

Current knowledge of factors influencing climate change literacy among nursing students has limitations. It is suggested that factors like student Grade Point Average (GPA), perceived teacher knowledge of climate change, healthy lifestyle habits, and socio-demographic backgrounds [35, 36] be considered. These factors are crucial for understanding various aspects of climate literacy, such as knowledge of climate science, health impacts, adaptation and mitigation strategies, and communication and advocacy skills [36] [33, 37, 38]

This study acknowledges the limited research on climate change literacy among Egyptian nursing students. The multi-site approach across various locations in Egypt will capture diverse perspectives and experiences. Recruiting a broad sample will also ensure robust and generalizable findings beyond specific institutions or regions. Ultimately, this research aims to identify the predictors of climate change literacy among nursing students in A Multi-Site Survey.

## Research questions

- What is the current level of climate change literacy among nursing students?
- What factors can be identified as predictors influencing the climate change literacy of nursing students?

## Methods

### Design and setting

This multi-sites descriptive, cross-sectional study followed a Consensus-Based Checklist for Reporting Survey Studies (CROSS) across all governorates of Egypt (*n* = 27) [39].

### Participants

We recruited the study sample from all the nursing students enrolled in 22 faculties and five institutes in Egypt to provide geographically representative samples of the country’s three major demographic regions: Middle Egypt, Upper Egypt, and Lower Egypt. This division described the country based on geographic and cultural differences.

**Fig. 1:**
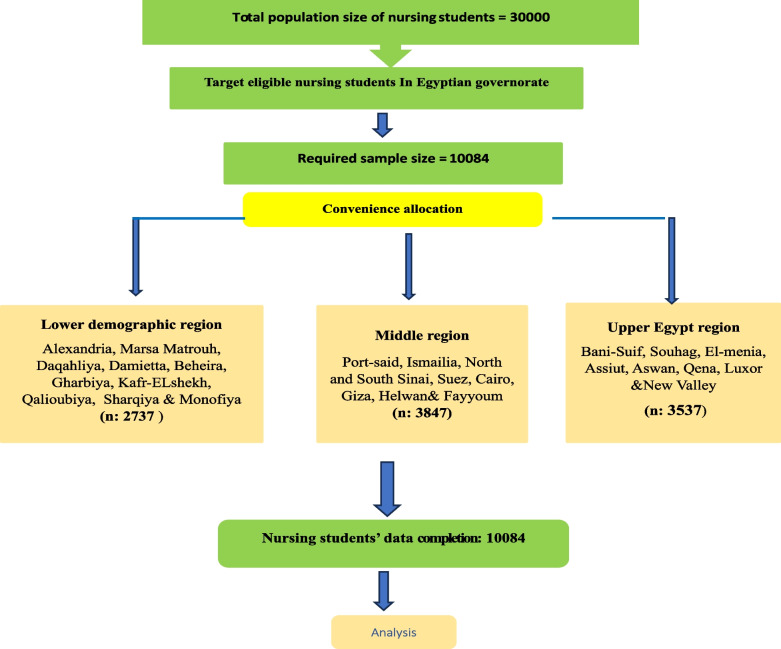
Sample flow graph

Open Epi, Version 3, was used to estimate the required sample size. The minimum sample size was 759 students, calculated based on a 95% confidence level, a 5% margin of error, a 50% response distribution, and a total population of nursing students in the studied setting of around 3000. Ten thousand eighty-four students responded to our questionnaire, representing about 33.6% of the study population. The survey link was made available to all students, reaching a potential audience of 30,000. However, only 10,084 students completed the questionnaire, representing approximately 33.6% of the study population. To prevent repeated participation, the survey system recorded IP addresses to ensure that each student could only submit the questionnaire once. Additionally, reminders were sent to participants who still needed to complete the survey, minimizing the likelihood of students forgetting their participation and filling out the survey again (Fig. 1).

The selection criteria were nursing students aged older than 18. We targeted nursing students who consented voluntarily to participate in this study and understood its purpose. Exclusion criteria were those who failed to complete the questionnaires and were unwilling to continue participating in the study for any reason.

Nursing education in Egypt spans five years, combining four academic years with a mandatory internship. The curriculum progresses from foundational sciences and basic nursing skills in the first year to specialized nursing care and leadership in the senior year. Throughout the program, students gain practical experience through clinical rotations, culminating in the internship year, where they apply their knowledge under the guidance of experienced nurses, preparing them for independent nursing practice.

### Instruments

In addressing the imperative of assessing climate change literacy among nursing students, a self-administered online survey served as the primary instrument for data collection. The researchers developed the ‘Predictors of Nursing Students’ Climate Change Literacy Scale’ to measure the factors influencing climate literacy among nursing students, identifying potential predictors based on existing literature [11, 31, 32]. It was designed to encompass an array of pertinent dimensions, facilitating a comprehensive investigation into the predictors of climate literacy among nursing students. This scale was developed into an English scale, then translated to Arabic, and then back-translated from Arabic to English by a qualified translator. Next, the translated and back-translated versions were compared against the original English scale. Corrections were made, and the translated version matched closely with the original English version. (Supplementary 1).

### Geographic diversity and socio-demographics

To capture the nuanced interplay of geographical and socio-demographic factors, the survey incorporated specific inquiries about the respondents’ regional affiliations, distinguishing between locales such as upper and lower regions and their urban or rural residences. Furthermore, sociodemographic characteristics like age, sex, income, and parental education that may shape nursing students’ perspectives on climate change were involved in this part of the survey.

### Student health habits and academic performance

The instrument probed into student health habits, encompassing dietary choices, physical exercise routines, sleep patterns, and chronic diseases or smoking habits. Concurrently, the academic performance section elucidated the participants’ academic level and overall GPA.

### Faculty knowledge and institutional support

Faculty dynamics and institutional backing were explored through a nuanced lens, with participants providing insights into faculty members’ perceived knowledge of climate change and the availability of resources dedicated to climate change education. The survey also sought to ascertain institutional policies or initiatives concerning climate change and health education. The survey also inquired into nursing students’ exposure to various climate change education methods, including lectures, case studies, simulations, field trips, and guest speaking. The frequency and depth of engagement with these methods and the student’s perceptions of their effectiveness added layers of understanding to integrating climate literacy within the academic sphere. This scale consisted of six statements, and students rated each statement against a point Likert scale ranging from one to five as 1 = strongly disagree, 2 = disagree, 3 = neutral, 4 = agree, and 5 = strongly agree. A clear categorization method was employed to ensure replicability when interpreting the item scores. Scores within the first third of the possible range (1.00 to 1.66 points) reflect a low level of agreement with the statement. Scores falling between one-third and two-thirds of the range (1.67 to 3.33 points) signify a moderate level of agreement, while scores in the last third (3.34 to 5.00 points) indicate a high level of agreement. This approach was selected to provide a nuanced interpretation across the full spectrum of possible scores.

### Domains of student climate literacy

Lastly, the instrument systematically assessed students’ proficiency in distinct domains of climate literacy, covering Climate Science, Climate Health Impacts, Adaptation and Mitigation Strategies, and Communication and Advocacy. Each domain encapsulated targeted questions evaluating the participants’ understanding of fundamental climate science principles, knowledge of health risks linked to climate change, awareness of adaptation and mitigation strategies, and the role of communication and advocacy in addressing climate-related challenges. The instrument consists of four domains, and each domain contains five statements that students rated against a point Likert scale from one to five as 1 = strongly disagree, 2 = disagree, 3 = neutral, 4 = agree, and 5 = strongly agree. The total scale score was 100 points. The item’s score interpreted according to the following items falling within the first third of the possible score range (1.00 to 1.66 points) indicates a low level of agreement with the statement. Items scores falling between one-third and two-thirds of the potential score range (1.67 to 3.33 points) indicate a moderate level of agreement with the statement, and items scores falling within the last third of the possible score range (3.34 to 5.00 points) indicate a high level of agreement with the statement [35].

Each tool domain consists of five items, with a score ranging from 5 to 25 points. Scores between 5.00 and 8.33 points indicate a low level of perceived knowledge. This suggests that students in this range may need a greater understanding of key concepts within the domain and would benefit from targeted learning to strengthen their knowledge. Scores between 8.34 and 16.66 points suggest a moderate level of perceived knowledge. At the same time, students in this range generally grasp the domain’s concepts, but they may still require additional learning to master the material fully. Finally, scores between 16.67 and 25.00 points reflect a high level of perceived knowledge, indicating that students in this range strongly understand the key concepts within the domain and have effectively mastered the material. This interpretation ensures that each score range is thoroughly explained and consistently tied to students’ perceived knowledge levels.

### Adaptation, validity, and reliability of the study instrument

Fifteen experts in nursing education, public health, and academic professionals in environmental psychology revised the tool “Predictors of Nursing Students’ Climate Change Literacy” to assess face validity for the tool completeness, item clarity, and the items’ relevance for the measured construct. The appropriate modifications were made accordingly.

### Measurements to ensure the validity and reliability of the tool

During the scale development, item validity and reliability analyses were conducted, including item-total correlations, Cronbach’s alpha for internal consistency, and content validity index (CVI) scores obtained from expert reviews. Additionally, pilot testing with 30 students was conducted to refine the scale.

CVI was done to ensure the survey content adequately covers the intended domain. This was done by fifteen experts in nursing education, public health, and academic professionals in environmental psychology**.** The item validity of adequacy, clarity, and relevance for each item ranged from 0.68 to 1. Experts’ content validity index (CVI) ranged from 0.84 to 0.94.

### Internal consistency testing

The researcher conducted a pilot study on 30 students as recommended for the sample pilot study, sampling from 10 to 40 participants per group [36]. The researcher conducted this pilot using an online survey of the tools. Students’ responses were used to test the internal consistency of the tools using Cronbach’s alpha reliability. All alpha values of the tools and their domains were above 0.75, which is acceptable.

While confirmatory factor analysis (CFA) was performed to assess construct validity, exploratory factor analysis (EFA) was not conducted as the survey was designed based on well-established constructs from existing literature [11, 31, 32]. CFA results indicated strong construct validity, with factor loadings exceeding 0.5 for all items.

We additionally conducted principal component analysis (PCA) to explore the underlying structure of the questionnaire. The PCA identified four distinct components for climate literacy with eigenvalues exceeding one, explaining a cumulative variance of 57.38% of the total variance. The faculty knowledge and support section analysis revealed a single component explaining 52.21% of the variance.

### Pilot study

A pilot study was carried out on 10% of all the nursing students being surveyed (*n* = 1009) who were chosen randomly. The pilot study appraised the time required to complete the instruments and tested their clarity, applicability, and possibility. No modifications were made, so the participants in the pilot study were included in this study.

### Data collection

The researchers created the questionnaire using Microsoft Office Forms, an online platform. This online data collection method was chosen to reach many students across all regions. Allowing students to fill out the questionnaire at home saved lecture time and provided them with the flexibility to complete the survey at their convenience. This approach also allowed students to focus more on their studies without the constraints of a classroom setting. Additionally, using an online format significantly reduced the cost for the researchers compared to printing and distributing paper surveys.

Each researcher shared the invitation link with the students in the institution where they are affiliated. Social media (Facebook, WhatsApp, and Telegram) and Microsoft Office team chat were used to invite the students. The invitation included a brief introduction of the research purpose and scope, the estimated time required to complete the survey, and a link to access the online questionnaire. To ensure that the nursing student fills out the survey, the researcher anonymously sends the link survey only to formal nursing student groups that contain only nursing students for more communication and to send study information. The researchers monitored the response rate and provided reminder messages every two weeks to encourage participation. Data was collected for five months, from the 1st of July 2023 to November 2023. The average time taken to complete the questionnaire was 25 min.

### Ethical consideration

The study protocol was approved by the ethical committees of the Faculties of Nursing, Damanhur University (Approval no; **85-d**). All necessary information about the study was introduced in the first section of the survey. The questionnaire included a statement related to the aim and nature of the study. All participants click on agree to give their informed consent before beginning their response to the online survey. The respondents were assured of the confidentiality and anonymity of their responses, the voluntary nature of their participation, and that non-participation did not affect their grades or lead to harmful consequences.

### Data analysis

The data collected for this study were analyzed using the Statistical Package for Social Science (SPSS) version 28. Categorical variables were represented as frequencies and percentages, offering a coherent representation of the distribution within each category. Simultaneously, continuous variables were characterized by mean and standard deviation (SD) values after confirming normality assumptions through the Kolmogorov–Smirnov test. Finally, SPSS AMOS is used to measure confirmatory factors regarding the developed tool.

Independent t-tests and one-way ANOVA tests were executed to ascertain relationships between variables and students’ climate literacy domains. The Pearson correlation coefficient was also employed to examine correlations between continuous variables and students’ climate literacy scores. Backward multiple linear regression was used to identify predictors associated with students’ climate literacy scores in each domain. Acknowledging that this method identifies correlations rather than establishing causality is important. The significance level (α) for all statistical tests was established at less than 0.05, signifying a threshold beneath which results were deemed statistically significant.

## Results

Table 1 represents characteristics of the nursing student cohort (*N* = 10,084), offering insights into their sociodemographic attributes, health behaviors, and academic standings. The demographic diversity indicates a relatively even distribution across upper (26.8%), middle (38.1%), and lower (35.1%) regions, while an equitable representation is observed between urban (50.4%) and rural (49.6%) locations. The mean age of students is 21.14 years (SD = 2.51), with a notable predominance of females (63.4%). Parental education reveals a diverse background, with 49.5% having parents with a Bachelor’s degree and 46.3% with a high school education or less. The mean household income is $453.80 (SD = $165.99). Health habits indicate that 69.4% do not maintain a well-balanced diet, 32.4% engage in physical exercise four times or more per week, and 78.7% sleep for 6–8 h. Academic performance distribution shows 30.2% in the intern category, and the overall grade point average is 2.71 (SD = 0.76).

**Table 1:** Description of students’ sociodemographic, health habits, and academic data (*N* = 10,084)

Students’ data		N (%) or mean (SD)
**Socio-demographics**		
Demographic diversity, N (%)	Upper region	2700 (26.8%)
	Middle region	3847 (38.1%)
	Lower region	3537 (35.1%)
Location, N (%)	Urban	5082 (50.4%)
	Rural	5002 (49.6%)
Age, mean (SD)		21.14 (2.51)
Sex, N (%)	Male	3694 (36.6%)
	Female	6390 (63.4%)
Parental education, N (%)	High school or less	4664 (46.3%)
	Bachelor’s degree	4993 (49.5%)
	Master’s degree or higher	427 (4.2%)
Average household income in dollar, mean (SD)		453.80 (165.99)
**Health status and habits**		
Eat a well-balanced diet, N (%)	Yes	3083 (30.6%)
	No	7001 (69.4%)
Practice physical exercise four times or more per week, N (%)	Yes	3269 (32.4%)
	No	6815 (67.6%)
Sleep from 6–8 h, N (%)	Yes	7941 (78.7%)
	No	2143 (21.3%)
Smoking, drink alcohol or other substance, N (%)	Yes	4101 (40.7%)
	No	5983 (59.3%)
Having chronic disease, N (%)	Yes	793 (7.9%)
	No	9291 (92.1%)
**Academic performance**		
Current academic level, N (%)	Freshman	844 (8.4%)
	Sophomore	2394 (23.7%)
	Junior	1469 (14.6%)
	Senior	2329 (23.1%)
	Intern	3048 (30.2%)
Overall grade point average (GPA), mean (SD)		2.71 (0.76)

Table 2 provides a detailed examination of faculty knowledge and support for climate literacy among nursing students. The perceived faculty members’ understanding of climate change and health education is reported with a mean score of 2.69 (SD = 1.02), suggesting a moderate level of perceived faculty expertise in this domain. The availability of climate change-related resources, such as textbooks and modules, is indicated by a mean score of 1.08 (SD = 0.29), reflecting a relatively low level of accessibility. Institutional support, as measured by the availability of policies and initiatives regarding climate change and health education, is reported with a mean score of 0.88 (SD = 0.30). The introduction of methods to integrate climate literacy into the nursing curriculum, including lectures (1.45, SD = 0.49), case studies (1.29, SD = 0.45), simulations (1.38, SD = 0.48), field trips (1.26, SD = 0.44), and guest speaking (1.28, SD = 0.45), demonstrates a diverse range of educational approaches. The frequency and depth of engagement with climate literacy practices are reported with a mean score of 1.68 (SD = 0.47), suggesting a moderate level of involvement. Lastly, the perceived effectiveness of climate literacy practices receives a mean score of 1.31 (SD = 0.46), indicating a generally low perception among students.

**Table 2:** The mean score of faculty knowledge and support for climate literacy (N: 10,084)

Faculty knowledge and support points	Mean (SD)
Perceived faculty members’ knowledge of climate change and health education	2 69 (1.02)
Availability of climate change-related resources such as textbooks and modules	1.08 (0.29)
Availability of institutional policies and initiatives regarding climate change and health education	0.88 (0.30)
Introduction of methods to integration climate literacy into the nursing curriculum	
Lectures	1.45 (0.49)
Case Studies	1.29 (0.45)
Simulations	1.38 (0.48)
Field trip	1.26 (0.44)
Guest speaking	1.28 (0.45)
Frequency and depth of engagement with climate literacy practices	1.68 (0.47)
Perceived effectiveness of climate literacy practices	1.31 (0.46)

Mean (SD) for rating score on a five-point Likert scale from 1 to 5

Table 3 elucidates the health literacy domains of nursing students within the context of climate change, offering a comprehensive examination of their knowledge and perceptions across distinct facets. In climate science, students exhibit a moderate understanding in the first domain, reflected in the overall mean score of 14.38 (SD = 2.06) out of a possible 25 points, with individual sub-scores ranging from 2.14 to 3.62. Moving to the second domain, climate health impacts, the mean score of 17.72 (SD = 2.11) indicates a high comprehension score, with individual sub-scores ranging from 3.21 to 3.99. However, the third domain, adaptation and mitigation strategies, reveals a mean score of 13.33 (SD = 2.07), suggesting a moderate level of understanding. Individual sub-scores range from 2.14 to 3.14. Finally, in the fourth domain, communication and advocacy, students demonstrate a moderate level of understanding, with a mean score of 14.41 (SD = 2.51). While there is variability in sub-scores ranging from 2.71 to 3.08, these results underscore a generally proficient grasp of climate-related health literacy among nursing students. Notably, each domain’s total score is 25, with the lowest possible score being 5, and each point represents a score out of five.

**Table 3:** The mean score of nursing students’ health literacy domain (*N*: 10,084)

Climate literacy domains	Mean (SD)
**Domain 1: Climate Science**	**14.38 (2.06)**
The Earth’s average temperature has been rising significantly in the past century	3.14 (0.78)
The leading cause of this rise in temperature is the increased concentration of greenhouse gases in the atmosphere	2.14 (0.74)
These greenhouse gases, like carbon dioxide and methane, trap heat from the sun, causing the planet to warm	2.69 (1.01)
Natural cycles like volcanic eruptions and solar activity can influence Earth’s climate, but human activities are the dominant driver of climate change	3.62 (0.88)
Positive feedback loops in the climate system, like melting ice reflecting less sunlight and releasing more methane from permafrost, can amplify the effects of climate change	2.78 (1.08)
**Domain 2: Climate Health Impacts**	**17.72 (2.11)**
Heatwaves, air pollution, and water scarcity, all linked to climate change, can have severe health consequences like heatstroke, respiratory problems, and infectious diseases	3.21 (0.93)
Specific populations, like children, older adults, and people with existing health conditions, are more vulnerable to climate-related health risks	3.99 (0.85)
Climate change can also disrupt ecosystems and food production, leading to malnutrition and food insecurity, with further health impacts	3.31 (0.98)
Nurses and healthcare professionals are crucial in identifying and managing climate-related health risks in their patients and communities	3.48 (1.08)
Effective communication and collaboration across sectors, including healthcare, are essential for building resilience and adapting to the health impacts of climate change	3.70 (0.79)
**Domain 3: Adaptation and Mitigation Strategies**	**13.33 (2.07)**
Reducing reliance on fossil fuels and transitioning to renewable energy sources like solar and wind power is crucial for mitigating climate change	3.14 (0.78)
Individual actions like using public transportation, reducing energy consumption at home, and adopting sustainable food choices can also contribute to climate change mitigation	2.14 (0.74)
Investing in green infrastructure, like drought-resistant crops and early warning systems for extreme weather events, can help communities adapt to the impacts of climate change	2.58 (0.98)
Climate-resilient healthcare systems should be prepared for increased heatwaves, floods, and other climate-related disasters to ensure continued access to essential healthcare services	2.93 (1.08)
Advocating for local, national, and international climate-friendly policies is essential to drive systemic change and accelerate the transition to a low-carbon future	2.53 (0.99)
**Domain 4: Communication and Advocacy**	**14.41 (2.51)**
I feel comfortable and confident explaining the link between climate change and health to patients and community members	2.86 (0.94)
I can effectively tailor my communication about climate change to different audiences, considering their knowledge, beliefs, and concerns	2.71 (1.27)
I promote climate action and advocate for climate-friendly policies in my community	2.94 (1.03)
I believe nurses and healthcare professionals are responsible for raising awareness about climate change’s health impacts and advocating for solutions	2.80 (1.17)
Social media and other communication tools can mobilize public support for climate action and policy change	3.08 (1.05)

Each sub-scale’s mean (SD) rating is 1 to 5 points for each item

Table 4 comprehensively explores the nuanced relationship between nursing students’ climate literacy scores, sociodemographic factors, and faculty climate change knowledge and support. Across sociodemographic categories such as demographic diversity, location, age, sex, parental education, and average household income, statistical tests revealed no significant differences in climate literacy scores for domains 1 to 4. This suggests a consistent distribution of climate knowledge across diverse student backgrounds. Health-related variables, including dietary habits, exercise, sleep patterns, substance use, and chronic diseases, also exhibited non-significant associations with climate literacy scores. Notably, a significant difference was identified in domain two scores across academic levels (*F* = 1.36, *p* = 0.02), indicating that sophomore students may have higher climate health impacts literacy. Furthermore, a positive correlation emerged between overall grade point average (GPA) and domain one scores (*r* = 0.62, *p* < 0.001), revealing that superior academic performance aligns with enhanced climate science literacy. Faculty factors significantly influenced students’ climate literacy, with perceived faculty members’ knowledge of climate change exhibiting positive correlations with all domains (*r* = 0.51 to 0.41, *p* < 0.001).

**Table 4:** Relationship between student climate literacy scores, students’ data, and faculty climate change knowledge and support scores

Variables	Test of significance with Domain 1	Test of significance with Domain 2	Test of significance with Domain 3	Test of significance with Domain 4
Socio-demographics				
Demographic diversity	F = 1.630, *p* = .196	F = .454, *p* = .635	F = 2.160, *p* = .115	F = 1.125, *p* = . .325
Location	t = .044, *p* = .602	t = .105, *p* = .073	t = -.246, *p* = .642	t = -.117, *p* = .985
Age	r = .009, *p* = .350	r = -.005, *p* = .651	r = .007, *p* = .464	r = .010, *p* = .301
Sex	t = 3.169, *p* = .075	t = 2.165, *p* = .141	t = 0.349, *p* = .554	t = 3.049, *p* = .081
Parental education	F = .578, *p* = .561	F = .128, *p* = .880	F = .250, *p* = .779	F = 1.207, *p* = .299
Average household income in dollar	r = .001, *p* = .913	r = .011, *p* = .283	r = -.008, *p* = .396	r = -.002, *p* = .817
Health status and habits				
Eat a well-balanced diet	t = .675, *p* = .326	t = .054, *p* = .736	t = .968, *p* = .329	t = .347, p = .949
Practice physical exercise	t = .104, *p* = .629	t = .448, *p* = .295	t = -.205, *p* = .977	t = -.902, *p* = .732
Sleep from 6–8 h	t = -.983, *p* = .325	t = -.259, *p* = .796	t = .814, *p* = .416	t = 2.112, *p* = .035*
Smoking, drinking alcohol, or others	t = -.829, *p* = .407	t = -.044, *p* = .965	t = -1.335, *p* = .182	t = .136, *p* = .892
Having chronic disease	t = .277, *p* = .782	t = .138, *p* = .890	t = -1.030, *p* = .303	t = .076, *p* = .939
Academic performance				
Current academic level	F = .934, *p* = .634	F = 1.363, *p* = .023*	F = .767, *p* = .927	F = 1.246, *p* = .080
Overall grade point average (GPA)	r = 0.62, *p* > .001**	r = .010, *p* = .317	r = .017, *p* = .094	r = -.015, *p* = .135
Faculty knowledge and support points				
Perceived faculty members’ knowledge of climate change	r = .507, *p* > .001**	r = .079, *p* > .001**	r = .034, *p* > .001**	r = .405, *p* > .001**
Availability of climate change-related resources	r = -.003, *p* = .775	r = -.003, *p* = .733	r = -.019, *p* = .058	r = .001, *p* = .940
Availability of institutional policies and initiatives	r = -.012, *p* = .213	r = .001, *p* = .966	r = -.012, *p* = .210	r = .010, *p* = .339
Methods to integration climate literacy into the curriculum				
Lectures	r = .005, *p* = .626	r = .008, *p* = .441	r = .006, *p* = .570	r = -.010, *p* = .310
Case studies	r = .002, *p* = .809	r = .018, *p* = .077	r = .000, *p* = .985	r = .003, *p* = .776
Simulations	r = -.011, *p* = .288	r = .004, *p* = .693	r = -.007, *p* = .511	r = -.004, *p* = .713
Field trip	r = .008, *p* = .411	r = -.011, *p* = .281	r = .005, *p* = .590	r = -.017, *p* = .082
Guest speaking	r = .004, *p* = .666	r = -.010, *p* = .293	r = -.006, *p* = .576	r = -.004, *p* = .680
Frequency and depth of engagement with faculty practices	r = .005, *p* = .581	r = -.005, *p* = .648	r = -.006, *p* = .569	r = -.004, *p* = .705
Perceived effectiveness of climate literacy practices	r = -.007, *p* = .512	r = -.004, *p* = .722	r = -.006, *p* = .553	r = .008, *p* = .913

t for independent sample t-test, r for person correlation, *F* for one-way-ANOVA test, *P* value less than 0.05 is significant*

Table 5 presents the results of backward multiple linear regression analyses, revealing factors associated with nursing students’ climate literacy scores across different domains. For students’ climate science scores, two significant predictors emerged: GPA (B = 0.08, 95% CI = 0.04–0.13, *p* < 0.001) and perceived faculty members’ knowledge of climate change (B = 1.03, 95% CI = 0.99–1.06,* p* < 0.001), indicating that a higher GPA and a positive perception of faculty knowledge are associated with enhanced climate science literacy. In the domain of climate health impacts scores, perceived faculty members’ understanding of climate change was a significant predictor (B = 0.16, 95% CI = 0.12–0.20, *p* < 0.001), signifying those students who perceive their faculty to be more knowledgeable tend to have higher climate health impacts literacy.

**Table 5:** Backward multiple linear regression for factors associated with students’ climate literacy

Dependent variable	Independent variables	B (95.0% CI)	S. E	t	*P*
**Climate science**	GPA	0.08 (0.04—0.13)	.023	3.636	< .001
	Perceived faculty knowledge of climate change	1.03 (0.99 -1.06)	.017	59.061	< .001
**Climate health impacts**	Perceived faculty knowledge of climate change	0.16 (0.12—0.20)	.021	7.910	< .001
**Adaptation strategies**	Perceived faculty knowledge of climate change	0.76 (0.72- 0.80)	.019	40.68	< .001
**Communication**	Sleep from 6–8 h	-0.13 (-0.25 to -0.01)	.061	-2.102	.036
	Perceived faculty knowledge of climate change	0.08 (.04—0.13)	.025	3.370	< .001

In this model, climate literacy domains are the dependent variables, while students’ sociodemographics, health habits, academic performance, and faculty knowledge and support are the independent variables. The results indicate associations between these factors and climate literacy domains; however, these findings should not be interpreted as evidence of causality. Additionally, outlier analysis and sensitivity tests were performed to confirm the robustness of the results

S.E. is the standard error, and a *P* value less than 0.05 is significant

Similarly, for students’ adaptation and mitigation strategies scores, perceived faculty members’ knowledge of climate change was a strong predictor (B = 0.76, 95% CI = 0.72–0.80, *p* < 0.001), highlighting the crucial role of faculty knowledge in developing students’ climate literacy. In the communication and advocacy domain, two factors influenced students’ scores. First, 6–8 h sleep duration was negatively associated with communication and advocacy scores (B = -0.13, 95% CI = -0.25 to -0.01, *p* = 0.036), suggesting that insufficient sleep might hinder students’ abilities in these areas. Second, perceived faculty members’ knowledge of climate change again emerged as a positive predictor (B = 0.08, 95% CI = 0.04–0.13, *p* = 0.001), indicating that students with a positive perception of their faculty’s climate knowledge tend to have higher communication and advocacy scores.

## Discussion

Nurses often profoundly understand the interconnections between environmental factors and public health, enabling them to play a pivotal role in promoting preventive measures and health education [33, 40]. However, there is a need for improved education and training specific to climate-related health challenges [12].

### Knowledge and support for climate literacy among nursing students

Our study subjects demonstrate moderate knowledge of climate change and health education and revealed support for integrating climate literacy into the nursing curriculum. This interaction employs various methods, such as lectures, case studies, simulations, field trips, and guest speaking, showcasing diverse educational approaches. AL Hussaini (2023) [41] suggests that educating students about the effects of climate change and the actions they can take to mitigate it is essential.

Additionally, Álvarez-Nieto and colleagues [9] discovered a growing positivity among nursing students regarding integrating sustainability and climate change topics into their curriculum.

Consistent with that, a study conducted by Ifegbesan and colleagues (2021) [42]found that most participants acquired information about climate change primarily from the Internet, social media, TV, and radio, with only a few obtaining such information from newspapers. This study also highlighted a negative and significant relationship between internet news and climate change awareness, with internet news emerging as the most concise set of predictors for climate change awareness [42].

Furthermore, the students’ exposure to different climates and environmental challenges in their respective locations may contribute to heightened global awareness of climate change [37]. The climate change events unfolding in Libya, particularly in Derna, the first east neighbor of Egypt, have served as a poignant reminder of the far-reaching impacts of climate change [38]. Last year, the eastern region of Libya was struck by Storm Daniel, an intense Mediterranean cyclone, causing an unprecedented natural disaster. The port city of Derna suffered severe damage, resulting in significant loss of life, destruction of infrastructure, and the displacement of thousands of residents [43].

As these climate-related incidents gain widespread media coverage [44] [2], nursing students in Egypt become increasingly attuned to the intersection of environmental factors and healthcare. Mass media plays a pivotal role in disseminating information about the consequences of climate change, creating a ripple effect that extends to educational institutions [6, 45]. The coverage prompts nursing students to recognize the importance of integrating climate considerations into their training, fostering a sense of responsibility towards the environment within the healthcare sector.

The consistent climate literacy observed among diverse nursing student backgrounds, without significant differences based on sociodemographic factors, suggests a uniform level of knowledge among Egyptian nursing students. This coherence may be attributed to standardized educational systems in Egypt, global accessibility of climate change information, a shared academic environment, a uniform curriculum that includes climate-related topics, and the perceived relevance of climate literacy to the nursing profession and societal concerns [46, 47]. However, it is essential to consider variations within this apparent uniformity, such as individual learning styles or exposure levels to environmental issues.

In contrast, a study conducted by Ifegbesan and colleagues in 2021 revealed noteworthy variations in climate change awareness based on both gender and the participants’ places of residence, as well as significant differences in climate change awareness among various educational and regional groups. While no significant difference in awareness was observed across different age groups, a significant positive correlation was identified between gender, place of residence, region, and climate change awareness [37].

### Nursing students’ health literacy domain

The current study indicates that nursing students demonstrate commendable comprehension of climate science and the health implications of climate change, along with a solid understanding of adaptation and mitigation strategies. This aligns closely with findings from İncesu and Yas [48], who observed that nursing students exhibited elevated levels of awareness concerning global climate change and demonstrated a strong foundation in climate science.

In our findings, nursing students from varied regions in Egypt exhibit a noteworthy grasp of climate science, climate health impacts, and adaptation knowledge. This outcome may be attributed to the nursing education program, as revealed in the result, which appears to prioritize and integrate a thorough coverage of climate-related topics, emphasizing the significance of environmental sustainability.

Moving to the second domain, climate health impacts indicate a high comprehension score. This aligns with Spante & Elf (2021) [15], who studied climate change and sustainability among nursing students, noting an understanding of the health implications of climate change but recognizing a challenge in translating this awareness into nursing practices.

The comprehensive nursing coursework covers environmental science, public health, and the interconnectedness of these fields. This multidisciplinary approach equips them with a broad knowledge base and the ability to comprehend complex issues [49]. Incorporating climate change topics into nursing curricula, using interdisciplinary methods, developing nursing practice abilities, and fostering advocacy and leadership skills are crucial. These strategies can prepare future nurses to tackle health issues linked to climate change. By empowering them to advocate for sustainable nursing practices and public health policies, these approaches ensure that nurses are well-equipped to address the health impacts of climate change [50].

Our participants revealed a moderate level of awareness regarding adaptation and mitigation strategies, as well as communication and advocacy. As for Aronsson and colleagues [39][32]studies, they conducted an integrative review exploring nursing students’ perspectives on climate change, revealing a divergence in awareness among students and recognizing nurses’ responsibility in climate change mitigation.

In addition, UNFCCC. (2023) [51], support for effective communication about climate change. It faces distinct challenges, including translating complex scientific terms into easily understandable language to convey climate impacts accurately. Additionally, there is a requirement to ensure that discussions about climate solutions are inclusive and accessible to a broad audience.

Williams et al. (2017). [52]stated that limited access to resources and information about climate change could hinder the development of robust adaptation and mitigation plans. Furthermore, there may be a need to prioritize climate change issues within the subjects’ communities or organizations, stemming from competing priorities, limited awareness, political and economic factors, institutional barriers, and a perception of limited impact [53].

### Predictors of climate literacy

Our study found that faculty knowledge about climate change is a powerful predictor of all domains of nursing students’ climate literacy, including climate science, climate health impacts, adaptation strategies, and communication. This finding highlights that nursing students are significantly influenced by the depth and breadth of their instructors’ understanding of climate change. When faculty members are well-versed in climate science, they are better equipped to integrate relevant information into their curricula, engage students in meaningful discussions, and emphasize the health impacts of climate change. Consequently, this enhanced instructional quality directly translates to higher climate literacy among nursing students, preparing them to address climate-related health issues in their future careers.

One possible explanation for this correlation is the role of faculty as primary sources of knowledge and role models for students. Faculty members with a robust understanding of climate change can effectively communicate its complexities and implications, making the subject more accessible and relevant to students. Additionally, knowledgeable faculty will likely incorporate contemporary research and real-world examples into their teaching, enriching the learning experience. This approach increases student engagement and helps students see the practical implications of climate change on health, fostering a deeper understanding and commitment to addressing these issues.

This finding implied that nursing faculty well-versed in climate science could help educational institutions enhance future healthcare professionals’ preparedness. This preparedness is crucial as climate change continues to pose severe public health risks, including increased incidence of diseases, extreme weather events, and environmental degradation. Nursing professionals knowledgeable about climate adaptation strategies can better advocate for and implement effective public health measures, ultimately improving community resilience against climate impacts. Consequently, investing in faculty development and climate education programs becomes a strategic priority for nursing schools. This finding could also give valuable insights to educators and policymakers. They might consider developing specialized training programs for nursing faculty to ensure they are well-informed about climate science and its health implications. Furthermore, nursing curricula could be updated to include comprehensive climate change education, emphasizing its relevance to public health and nursing practice.

Supporting studies corroborate this finding. For instance, a survey by Leffers et al. (2017) [54] emphasized the importance of integrating climate change content into nursing curricula to prepare students for future challenges. Also, the study of Kolenatý et al. (2022) [55] highlights the critical role of faculty in enhancing environmental health literacy among nursing students. It shows that nursing educators with comprehensive knowledge of environmental health and climate change are more effective in integrating these topics into the curriculum, thereby improving students’ literacy and awareness. In addition, the report of Neal-Boylan et al. (2019) [56]underscores the importance of healthcare professionals, including nurses, being well-versed in climate change to address its health impacts. The findings suggest that educators’ knowledge significantly influences students’ understanding and readiness to tackle climate-related health issues. Likewise, Mills (2022) [57]explored various educational strategies to enhance climate change literacy among nursing students. It identifies faculty expertise and engagement as critical factors, demonstrating that those knowledgeable faculties are more successful in delivering comprehensive and impactful climate education.

On the other hand, Limaye et al. (2020) [58] found that despite increased climate change education efforts, there still needs to be a significant gap in the practical application of this knowledge among healthcare students. Also, DeCamp (2024) [59]suggested that while faculty knowledge is essential, it may not be the sole determinant of student outcomes. Institutional support, student motivation, and resource access also play critical roles. Furthermore, Incesu and Yas (2024) [48] argued that while faculty knowledge is essential, integrating climate change education into various aspects of the curriculum and using interdisciplinary approaches are critical for adequate climate literacy. This perspective suggests that institutional support and curriculum integration might be as important or more essential than faculty knowledge alone. These discrepancies indicate that while faculty knowledge is crucial, other factors such as curriculum design, institutional support, and experiential learning opportunities also play vital roles in effectively educating nursing students about climate adaptation strategies.

The perception of faculty members’ knowledge of climate change significantly influences nursing students’ climate health impacts literacy by fostering a positive and engaging learning environment. This perception is linked to effective teaching methods, the role of faculty as positive role models, and the relevance of climate science to nursing practice. Students who view their instructors as knowledgeable are likelier to participate actively, trust the information presented, and develop a deeper understanding of climate-related health issues. Dong et al. (2023) [60]also highlight the broader impact of climate on cognitive performance, suggesting that future climates may exacerbate academic challenges. The predictive relationship between perceived faculty knowledge and students’ climate health impact scores suggests that faculty expertise and its perception are critical in shaping students’ engagement with and comprehension of climate health impacts (Ofori et al., 2023) [34]. This finding underscores the importance of investing in faculty development programs that enhance climate science knowledge and teaching effectiveness. By doing so, educators can better equip students to address climate-related health challenges in their future nursing practice, leading to more informed and proactive healthcare professionals.

Interestingly, our study revealed that the GPA of nursing students is a powerful predictor of their awareness of climate science, suggesting a strong correlation between academic performance and climate literacy. This finding implies that students who excel academically better understand and understand climate science. One possible explanation for this correlation is that students with higher GPAs often possess strong critical thinking skills, effective study habits, and a remarkable ability to assimilate complex information, all crucial for comprehending multifaceted issues like climate change. Additionally, these students might be more motivated and engaged in their studies, leading them to seek out and understand information beyond the primary curriculum.

This finding could give valuable insights into nursing education, suppose GPA is a reliable predictor of climate science awareness. In that case, nursing programs might consider using GPA as one of the criteria for identifying students who could benefit from or excel in specialized training or courses focused on climate change and health. Moreover, this correlation underscores the importance of fostering strong academic skills in nursing students, as these skills contribute to their overall educational success and ability to understand and address critical global health issues. It also suggests that improving students’ academic performance might enhance their climate science literacy.

Supporting studies further validate the connection between academic performance and subject-specific literacy. For instance, a survey by Belton and Brinkmann (2024) [61]found that students with higher educational achievements were more likely to understand environmental issues better. Similarly, research by Erguvan (2024) [62] and El-Sayed & Abdelaliem (2023) [63] indicated that students who perform well academically are generally more adept at integrating and applying knowledge across different domains, including climate science. These studies support the idea that academic excellence can significantly influence students’ ability to grasp complex, interdisciplinary topics like climate change.

Contradictory studies suggest that GPA might not be climate science awareness’s sole or primary determinant. Talwar et al. (2023) [64] research on climate change education emphasized the role of experiential learning and interdisciplinary approaches over traditional academic performance metrics. Additionally, Bist and Mayberry (2022) [65]highlight the importance of engaging and relevant content in fostering climate literacy, pointing out that students’ interest and personal engagement with the material can be more critical than their GPA [65]. These studies suggest that while GPA is a helpful indicator, other factors such as teaching methods, curriculum design, and student engagement are also crucial in determining climate science awareness.

## Strengths & limitations

Our study contains a large sample size, comprising 10,084 nursing students from all 27 governorates in Egypt, enhancing our findings’ statistical power and robustness. Our study highlights significant predictors of climate change literacy and the valuable nursing students who advocate climate change.

However, the focus on nursing students from a single country, Egypt, may need to be revised to allow the generalizability of our findings to nursing students in other countries with different cultural, educational, and environmental contexts. Additionally, using a developed climate literacy scale must validate tools across various cultural and academic contexts to ensure comprehensive insights into predictors of climate change literacy among nursing students internationally.

## Implications

In integration with our main findings, several implications for nursing practice, health policy, education, and research can be drawn:

### Nursing practice

The high level of knowledge of climate health impacts suggests that nursing practitioners can play a pivotal role in educating patients and communities about the health effects of climate change. Nursing professionals knowledgeable about climate adaptation strategies can better advocate for and implement effective public health measures, ultimately improving community resilience against climate impacts.

### Education

Nursing education curricula could be updated to include comprehensive climate change education, emphasizing its relevance to public health and nursing practice. It is crucial to provide extensive training to empower nursing faculty to incorporate climate change curriculum into their courses. As influential figures in the healthcare system, the faculty will be able to champion policy changes that promote environmental sustainability and effectively educate students on the link between climate change and healthcare. Equipping them with the necessary materials and skills through this program is vital. Additionally, it is essential to keep faculty members up to date with the latest findings and effective advocacy strategies against climate change through continuous professional development opportunities. By investing in the training and development of nursing faculty, educational institutions can prepare future generations of nurses to effectively address climate change issues and contribute to creating a healthier, more sustainable world.

### Health policy

The findings underscore the need for health stakeholders to incorporate climate health knowledge into public health frameworks. In addition, given the significant role of perceived faculty knowledge, policies should support ongoing education and training for nursing faculty on climate change to ensure they can effectively teach and advocate for climate literacy.

### Research

Additional research is needed to explore the factors influencing climate change literacy among academic students in other regions and among other healthcare professionals. Research should evaluate the effectiveness of various educational interventions to improve climate change literacy among nursing students and faculty. Conduct a longitudinal study or incorporate a follow-up phase to gain a deeper understanding of changes in climate change literacy over time and causality. Research highlights the necessity of a comprehensive approach to data collection and analysis, ensuring the validity and reliability of the findings, particularly when integrating self-reported data with objective measures such as knowledge assessments or faculty evaluations.

## Conclusion

It can be concluded that nursing students possess a moderate understanding of climate science, communication, advocacy skills, and knowledge of adaptation and mitigation strategies, with the highest level of expertise in climate health impacts. The study highlights that climate literacy across diverse student backgrounds is the same. Significantly, the perceived knowledge of faculty on climate change correlates with higher climate literacy among students and serves as a critical predictor. These findings underscore the need to integrate comprehensive climate change education into nursing curricula and support faculty development to enhance climate literacy. Addressing these areas is crucial for preparing future nurses to manage and mitigate climate change’s health impacts effectively.

## Supplementary Information

Supplementary Material 1.

## Data Availability

Yes, I have research data to declare.”The datasets used and analyzed during the current study are available from the corresponding author on reasonable request.”

## Data Availability

The datasets used and analyzed during the current study are available from the corresponding author on reasonable request.
