# Supplementary material for: Predictors of climate change literacy in the era of global boiling: a cross-sectional survey of Egyptian nursing students
Source: BMC Nurs. 2024 Sep 26;23:676. doi: 10.1186/s12912-024-02315-y (PMC11425957; doi:10.1186/s12912-024-02315-y)
Supplement: Supplementary file 1 — Supplementary Material 1. [file 12912_2024_2315_MOESM1_ESM.docx]

**Supplementary 1: Predictors of Nursing Students’ Climate Change Literacy tool**

1. **Socio-demographics**

- **Demographic diversity:………….**
- **Location:………….**
- **Age, mean :………….**
- **Sex:………….**
- **Parental education:………….**
- **Average household income in dollar:………….**

1. **Health status and habits**

- **Eat a well-balanced diet:**
- **Practice physical exercise four times or more per week:**
- **Sleep hours:**
- **Smoking, drink alcohol or other substance:**
- **Having chronic disease:**

1. **Academic performance**

- **Current academic level:**
  - **Freshman**
  - **Sophomore**
  - **Junior**
  - **Senior**
  - **Intern**
- **Overall grade point average (GPA):**

1. **Faculty knowledge and support points**

- **Perceived faculty members' knowledge of climate change and health education**
- **Availability of climate change-related resources such as textbooks and modules**
- **Availability of institutional policies and initiatives regarding climate change and health education**
- **Introduction of methods to integration climate literacy into the nursing curriculum**
- **Lectures**
- **Case Studies**
- **Simulations**
- **Field trip**
- **Guest speaking**
- **Frequency and depth of engagement with climate literacy practices**
- **Perceived effectiveness of climate literacy practices**

1. **Climate literacy domains**

**Domain 1: Climate Science**

- **The Earth's average temperature has been rising significantly in the past century.**
- **The main cause of this temperature rise is the increased concentration of greenhouse gases in the atmosphere.**
- **These greenhouse gases, like carbon dioxide and methane, trap heat from the sun, causing the planet to warm.**
- **Natural cycles like volcanic eruptions and solar activity can also influence Earth's climate, but human activities are the dominant driver of current climate change.**
- **Positive feedback loops in the climate system, like melting ice reflecting less sunlight and releasing more methane from permafrost, can amplify the effects of climate change.**

**Domain 2: Climate Health Impacts**

- **Heatwaves, air pollution, and water scarcity, all linked to climate change, can have severe health consequences like heatstroke, respiratory problems, and infectious diseases.**
- **Certain populations, like children, the elderly, and people with existing health conditions, are more vulnerable to climate-related health risks.**
- **Climate change can also disrupt ecosystems and food production, leading to malnutrition and food insecurity, with further health impacts.**
- **Nurses and healthcare professionals have a crucial role in identifying and managing climate-related health risks in their patients and communities.**
- **Effective communication and collaboration across sectors, including healthcare, are essential for building resilience and adapting to the health impacts of climate change.**

**Domain 3: Adaptation and Mitigation Strategies**

- **Reducing our reliance on fossil fuels and transitioning to renewable energy sources like solar and wind power are crucial for mitigating climate change.**
- **Individual actions like using public transportation, reducing energy consumption at home, and adopting sustainable food choices can also contribute to climate change mitigation.**
- **Investing in green infrastructure, like drought-resistant crops and early warning systems for extreme weather events, can help communities adapt to the impacts of climate change.**
- **Climate-resilient healthcare systems should be prepared for increased heatwaves, floods, and other climate-related disasters to ensure continued access to essential healthcare services.**
- **Advocating for climate-friendly policies at local, national, and international levels is essential to drive systemic change and accelerate the transition to a low-carbon future.**

**Domain 4: Communication and Advocacy**

- **I feel comfortable and confident explaining the link between climate change and health to patients and community members.**
- **I can effectively tailor my communication about climate change to different audiences, considering their knowledge, beliefs, and concerns.**
- **I am engaged in promoting climate action and advocating for climate-friendly policies in my community.**
- **I believe nurses and healthcare professionals have a responsibility to raise awareness about the health impacts of climate change and advocate for solutions.**
- **Social media and other communication tools can be effectively used to mobilize public support for climate action and policy change.**
